# The burden of hospital-attended influenza in Norwegian children

**DOI:** 10.3389/fped.2022.963274

**Published:** 2022-09-07

**Authors:** Håkon Bøås, Terese Bekkevold, Lise Beier Havdal, Anne-Marte Bakken Kran, Astrid Elisabeth Rojahn, Ketil Størdal, Sara Debes, Henrik Døllner, Svein Arne Nordbø, Bjørn Barstad, Elisebet Haarr, Liliana Vázquez Fernández, Britt Nakstad, Truls Michael Leegaard, Olav Hungnes, Elmira Flem, Norwegian Enhanced Pediatric Immunisation Surveillance (NorEPIS) Network, Håkon Bøås

**Affiliations:** ^1^Norwegian Institute of Public Health, Oslo, Norway; ^2^Department of Paediatric and Adolescent Medicine, Akershus University Hospital, Nordbyhagen, Norway; ^3^Department of Microbiology, Oslo University Hospital, Oslo, Norway; ^4^Department of Paediatrics, Oslo University Hospital, Oslo, Norway; ^5^Department of Pediatrics, Østfold Hospital, Grålum, Norway; ^6^Division of Paediatric and Adolescent Medicine, Institute of Clinical Medicine, University of Oslo, Oslo, Norway; ^7^Department of Medical Microbiology, Østfold Hospital, Grålum, Norway; ^8^Department of Pediatrics, St. Olavs University Hospital, Trondheim, Norway; ^9^Department of Clinical and Molecular Medicine, Norwegian University of Science and Technology, Trondheim, Norway; ^10^Department of Medical Microbiology, St. Olavs University Hospital, Trondheim, Norway; ^11^Department of Paediatric and Adolescent Medicine, Stavanger University Hospital, Stavanger, Norway; ^12^Department of Medical Microbiology, Stavanger University Hospital, Stavanger, Norway; ^13^Department of Microbiology and Infection Control, Akershus University Hospital, Nordbyhagen, Norway; ^14^Division of Medicine and Laboratory Sciences, Institute of Clinical Medicine, University of Oslo, Oslo, Norway

**Keywords:** hospital-attended influenza, pediatric, children, surveillance, disease burden

## Abstract

**Background:**

Norwegian health authorities do not recommend universal pediatric vaccination against seasonal influenza. We aimed to estimate the incidence of influenza by age and underlying medical conditions in hospitalized Norwegian children aged <18 years.

**Methods:**

Active surveillance for influenza in children <18 years was implemented in five hospitals during 2015–18. Children with respiratory symptoms and/or fever were prospectively enrolled and tested for influenza. Surveillance data were linked to health registry data to estimate the national burden of influenza in hospitals.

**Results:**

In 309 (10%) out of 3,010 hospital contacts, the child tested positive for influenza, corresponding to an average incidence of 0.96 hospital-attended influenza cases per 1,000 children <18 years of age. Children <1 year of age (3.8 per 1,000 children) and children with underlying medical conditions (17 per 1,000 children with bronchopulmonary dysplasia) had the highest average incidence. Among <1 year old children, 3% tested positive for influenza, compared to 25% for children aged 6–17. Few children were vaccinated against influenza.

**Conclusions:**

Children <1 year of age and children with underlying medical conditions had a higher incidence of influenza requiring hospital treatment compared to the general population. Effective interventions against seasonal influenza for children in Norway should be considered.

## Introduction

Influenza is an important cause of acute respiratory illness in children (1–6), and, prior to the SARS-CoV-2 pandemic, 10% of childhood respiratory hospitalizations worldwide were attributed to influenza (3, 7–9). In the United States, the annual incidence of influenza hospitalizations in children aged <6 months has been reported to be 2.7– 9.1 per 1,000 children (3, 9, 10), compared to ~0.6 or fewer cases per 1,000 children <18 years of age (11). Similar rates have been reported for children <18 years in Norway, where a registry-based study estimated that 1.7% of the Norwegian population seeks primary healthcare due to influenza each season, with about 0.3 influenza-associated hospitalizations per 1,000 people <20 years of age. The highest rates (~0.6 per 1,000 children) are reported in children <5 years of age (4). Although children play a leading role in the transmission of seasonal influenza (12, 13), the knowledge of the influenza burden in children is limited (14, 15).

Most fatal influenza cases among children happen in developing countries (1). However, in the United states, 113 pediatric deaths were reported, on average, each season between 2010 and 2016 (16), often occurring before hospital admission or even healthcare treatment was sought (16, 17). About half of the influenza-related deaths occurred among children with no pre-existing medical conditions, with the highest rates among infants <6 months old (16, 17). In Norway, influenza-associated pediatric deaths are also reported almost every season (4).

Infants and children with pre-existing risk conditions have an increased risk of hospitalization (4, 5, 18) from influenza. They need management in an intensive care unit more often (19, 20), have an increased risk of bacterial pneumonia (19), and have a greater risk of influenza-related death (17). A recent Norwegian study found that immunocompromised children and children with epilepsy had the highest risk of hospitalization with influenza, followed by children with heart disease and lung disease (5). Among children hospitalized with influenza, 25% had one or more pre-existing risk factors for severe influenza, compared to 5% in the general population under 18 years of age (5). According to the current Norwegian recommendations, only children with risk conditions are recommended to receive annual vaccination against influenza (21–23), and coverage in the pediatric population is low (23). Therefore, a better understanding of the relative burden of influenza in the pediatric population is needed to refine the national influenza vaccine recommendations.

To this end, we aimed to measure the incidence of influenza in children <18 years of age who were admitted for outpatient or inpatient hospital treatment in Norway. The study was conducted by the Norwegian Enhanced Pediatric Immunization Surveillance (NorEPIS) network, established in 2014. NorEPIS implemented active hospital surveillance for acute respiratory and gastrointestinal infections in patients <18 years of age, engaging five hospitals constituting the catchment area for ~40% of all Norwegian children.

## Materials and methods

Following the standard of the European Center for Disease Control (ECDC) (24), and the Norwegian influenza surveillance (25), the influenza season was defined as the period from week 40 until week 20 the following year. From 2015 to 2018, influenza surveillance was implemented annually from week 40 to week 20 the following year, except for the year 2015 when surveillance was begun in week 49. Children <18 years of age referred to the hospital with respiratory symptoms and/or fever were prospectively enrolled. When a participant had several re-admissions caused by the same disease episode it was counted as one episode, and only the first hospital contact was used. Clinical data and information about healthcare use were collected using a standardized questionnaire, by medical record review, and *via* patient and/or caregiver interview. A detailed description of the inclusion and exclusion criteria is provided in the Supplementary Table 5.

### Sample collection

Nasopharyngeal flocked swabs or aspirates were collected by a hospital doctor or nurse from all enrolled patients within 72 h of the patients' arrival at the hospital. The samples were placed immediately into transport media and sent for analysis of influenza virus in the hospital laboratory, using in-house real-time polymerase chain reaction. Samples from four of the five hospitals were sent to the national reference laboratory for influenza at the Norwegian Institute of Public Health, where all positive samples, as well as 10% of negative samples, were retested and genotyped to identify viral subtype and lineage as well as to verify the validity of the hospital analyses.

### Registry data sources

Data were linked to national health registries using unique personal identification numbers. The Norwegian Patient Registry (NPR) and the Norwegian Control and Payment of Health Reimbursements (KUHR) Database together cover all government-funded health care in Norway. NPR contains information about all hospital contacts in Norway, including the International Classification of Diseases (ICD-10) diagnoses (26). The KUHR database—contains the International Classification of Primary Care (ICPC-2) diagnoses from all publicly funded general practitioners and primary care emergency clinics (27). All diagnoses assigned to the study participants during 2014–2019 were retrieved as well as the duration of hospital stays, date and time of arrival and discharge, and other variables (Supplementary Table 2). To identify underlying risk groups, all ICD-10 and ICPC-2 codes (Supplementary Table 1) assigned to study participants between 2008 and 2019 were retrieved.

Furthermore, we obtained the total number of hospital contacts during the 2015–2018 seasons in all Norwegian children <18 years of age, with any or a combination of the influenza ICD-10 codes J09–J11.1 as discharge diagnoses.

We collected additional information from the Medical Birth Registry of Norway (28) about prematurity, congenital malformations, and birth disorders (Supplementary Table 2.)

Information about the influenza vaccination status was collected from the Norwegian Immunization Registry SYSVAK (29). A valid vaccination was defined as a vaccine administered in the same influenza season as the recruitment season and more than 2 weeks before the start of symptoms. If the date of symptom onset was missing, the sampling date was used.

Background data on the population size in the hospital catchment areas, stratified by age, were retrieved from Statistics Norway (30).

### Risk groups

Children were considered to have high risk for influenza-associated hospitalization if they were born prematurely (gestational age <37 weeks) or had been previously diagnosed with underlying conditions such as trisomy 21, congenital heart disease, asthma, bronchopulmonary dysplasia, other pulmonary disease, neuromuscular disease, immunosuppression, epilepsy, and cancer. Children with these conditions were identified through the Medical Birth Registry of Norway, or through ICD-10 codes in the NPR and ICPC-2 codes in the KUHR database (Supplementary Table 1).

### Statistical analyses

Statistical analysis was performed in Stata version 15 (StataCorp LLC, College Station, Texas, US). We compared the percentage of influenza-positive and negative samples using a logistic regression model. To account for potential differences among the hospitals in the recruitment procedure and the seasonality of influenza, as well as potential differences in the age distribution among different groups, we included hospital, study season, month of hospital contact, and age group as independent variables in adjusted analyses.

The analysis involved only patients with documented symptom onset in the 10 days prior to receiving hospital treatment. If a patient had more than one hospital visit within 21 days of the same disease episode, only the first hospital encounter was used. The length of stay between influenza-positive and negative children was compared using the Wilcoxon rank-sum test.

### Estimates of influenza-related disease episodes

The total number of hospital-attended influenza cases was estimated using the proportions of influenza-coded participants with laboratory-confirmed influenza and the proportion of influenza-confirmed participants assigned ICD-10 influenza codes. Following the same procedure as outlined in Havdal et al. (31), these proportions were applied to the total numbers of influenza-coded hospitalizations in children <18 years of age registered in Norway during the study period, which were retrieved from the NPR for both the NorEPIS study catchment area and for the entire country.

### Estimate of age-specific influenza infections

The upper and lower boundaries of the incidence rates were derived by using the boundaries of the 95% confidence interval (CI) from the proportions of the estimated number of influenza episodes. Age-specific estimates were calculated by multiplying the total number of reported hospital contacts from the NPR by the proportion of cases in the various age groups among NorEPIS participants. The estimated mean annual number of national influenza-related hospital-attended children with underlying risk conditions was derived by taking the mean number of estimated national influenza cases multiplied by the proportion of influenza-positive NorEPIS participants with risk factors.

### Ethics

Written informed consent was obtained from both parents and legal guardians for children <16 years of age or from the patient if between 16 and 18 years of age. The study was reviewed and approved by the Regional Committees for Medical and Health Research Ethics, South-East A (2015/956).

## Results

**Figure 1:**
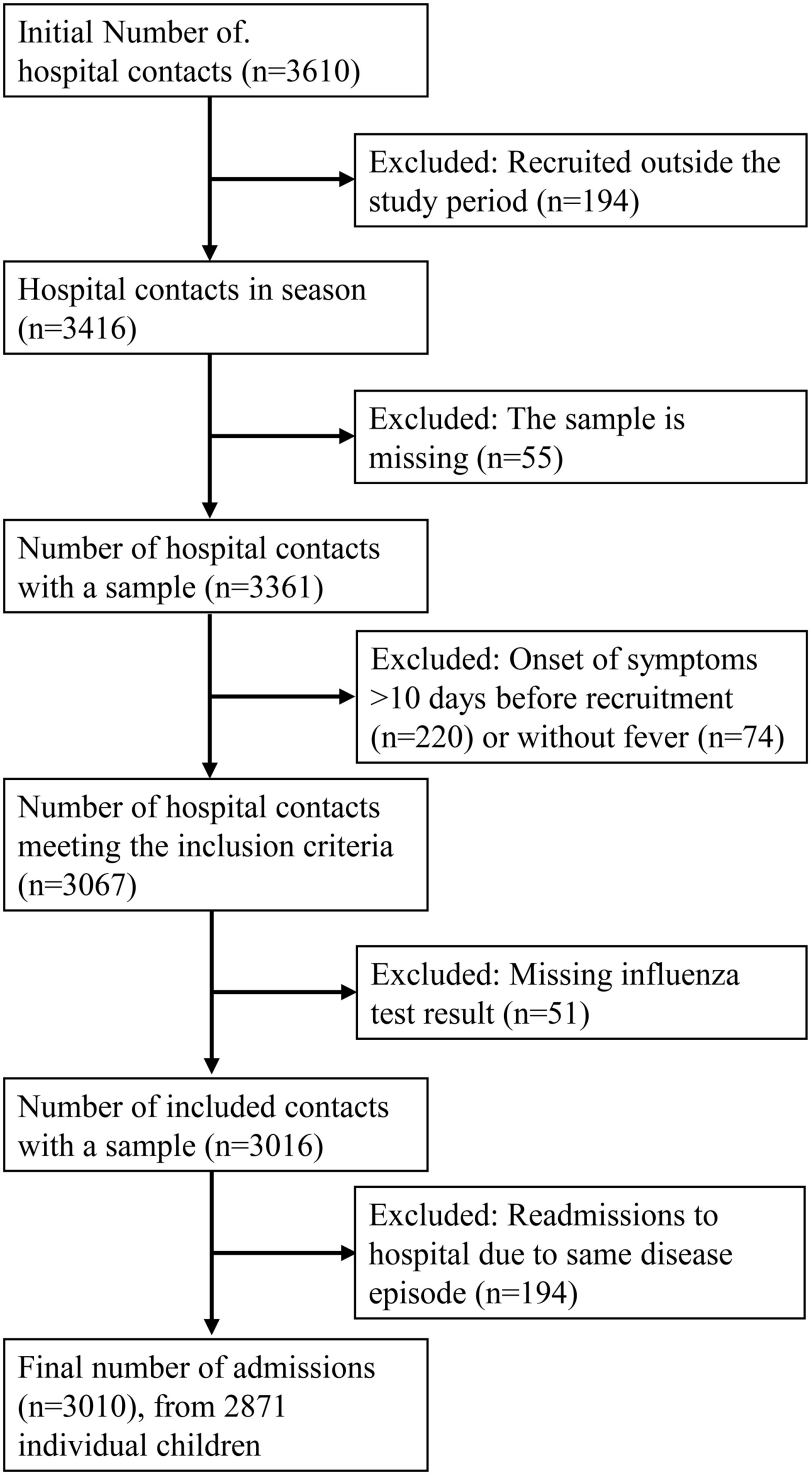
Flowchart of the recruitment process.

From December 2015 to May 2018, 3,391 individual participants with 3,610 hospital contacts were included in the study. After excluding participants recruited outside the surveillance period, re-admissions caused by the same disease episode, and participants who otherwise did not fulfill the inclusion criteria, 2,871 children with 3,010 hospital contacts were included in the analysis (Figure 1).

Of the 3,010 hospital contacts, 10.3% of all patients and 8.6% of inpatient cases tested positive for influenza (Table 1). Children <5 years of age accounted for almost 92% of all hospital contacts, and ~50% of the hospital contacts occurred in children <1 year of age. The probability of testing positive for influenza increased with age, and children aged 6–17 years presenting at the hospital for respiratory symptoms or fever were more likely to test positive for influenza compared with children <6 months of age. There were fewer female (42.7%) than male participants, with no significant association detected between sex and influenza infection. Similarly, among influenza-positive children, no significant association between sex and admittance as an inpatient was observed (Tables 1, 4).

**Table 1:** Total number of enrolled children, with logistic regression comparing influenza positive and negative children within categories.

	**Total number children tested**	**Number with positive influenza PCR-test**	**OR (95% CI)**	***P***
	**for influenza *N* = 3,010**	**by variable category *N* = 309 (%)**		
**Age group**
<6 m	978 (32.5)	30 (3.1)	Ref.	
6–11 m	469 (15.6)	34 (7.3)	2.47 (1.49–4.09)	<0.001
1–2 y	1,083 (36.0)	134 (12.4)	4.46 (2.97–6.70)	<0.001
3–5 y	234 (7.8)	49 (20.9)	8.37 (5.17–13.54)	<0.001
6–17 y	246 (8.2)	62 (25.2)	10.65 (6.70–16.93)	<0.001
Total	3,010 (100)	309 (10.3)		
**Sex**
Male	1,724 (57.3)	176 (10.2)	Ref.	
Female	1,286 (42.7)	133 (10.3)	1.01 (0.80–1.29)	0.91
Total	3,010 (100)	309 (10.3)		
**Hospital**
Oslo University Hospital, Ullevål	1,105 (36.7)	104 (9.4)	Ref.	
Akershus University Hospital	540 (17.9)	61 (11.3)	1.23 (0.88–1.71)	0.23
Østfold Hospital Kalnes	648 (21.5)	86 (13.3)	1.47 (1.09–2.00)	0.01
St. Olavs hospital	301 (10.0)	23 (7.6)	0.80 (0.50–1.28)	0.34
Stavanger University Hospital	416 (13.8)	35 (8.4)	0.88 (0.59–1.32)	0.55
Total	3,010 (100)	309 (10.3)		
**Outpatient or inpatient**
Outpatient	1,186 (40.3)	156 (13.2)	Ref.	
Inpatient	1,757 (59.7)	151 (8.6)	0.62 (0.49–0.79)	<0.001
Total	2,943 (100)	307 (10.4)		
**Influenza vaccinated**
Not vaccinated	2,970 (98.7)	304 (10.2)	Ref.	
Vaccinated	40 (1.3)	5 (12.5)	1.25 (0.49–3.22)	0.64
Total	3,010 (100)	309 (10.3)		
**Study season**
2015/2016	1,041 (34.6)	146 (14.0)	Ref.	
2016/2017	1,203 (40.0)	76 (6.3)	0.41 (0.31–0.55)	<0.001
2018/2019	766 (25.4)	87 (11.4)	0.79 (0.59–1.04)	0.10
Total	3,010 (100)	309 (10.3)		

Only 34 of the 2,871 (1.2%) children enrolled in the study were vaccinated against influenza before enrollment. Of these, five patients (12.5%) tested positive for influenza. Some of these children had multiple hospital visits during the study period and 40 of 3010 (1.3%) hospital contacts were made by vaccinated children. There was no significant association between testing positive for influenza and vaccination status (Table 1).

We compared children testing positive for influenza with influenza-negative children who had other respiratory infections. Children with influenza were significantly less likely to be admitted for inpatient hospital treatment compared to children testing negative for influenza (Table 1), also after including age as an independent variable [OR 0.68 (0.53–0.87), *P* <0.001]. Nearly one-half of the children with confirmed influenza (49.2%) were treated as inpatients (Table 1, Supplementary Table 2), with no statistically significant difference in the median length of stay [41 h, interquartile range (IQR) 19–98 h] compared to inpatients admitted for other reasons (48 h IQR 22–96 h, *p* = 0.16].

**Figure 2:**
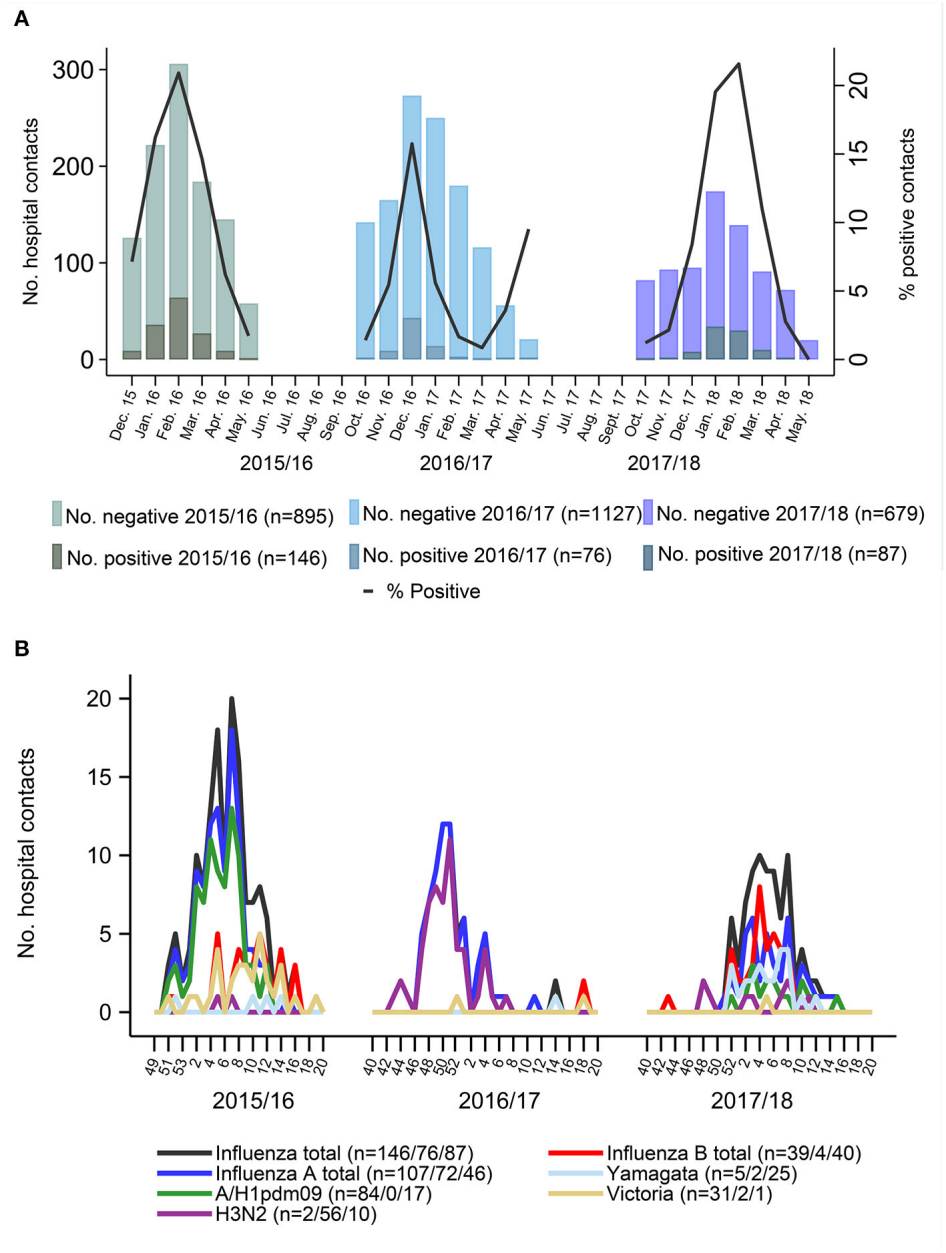
**(A)** Number of Influenza tested participants, and positivity rate through three influenza seasons. **(B)** Occurrence of influenza subtypes throughout three surveillance seasons. Black lines are the sum of all influenza cases per week. Blue lines are the sum of all influenza a cases and red line is the sum of all influenza b cases. Not all cases were fully typed due to shortage of material or low viral titers.

The largest number (47 %) of influenza cases were recruited during the first study season, whereas most of the study participants were enrolled during the second season, reflecting the variable seasonal load of influenza (Table 1; Figure 2). At peak season, between 15.8 and 20.9% of enrolled children were positive for influenza (Figure 2A). There was considerable variation between the circulating viral strains and genotypes detected in different seasons. While the 2015–2016 season was dominated by influenza A/H1pdm09 with some B/Victoria circulating during the second half of the season, the 2016–2017 season was almost exclusively dominated by A/H3N2. Both A/H1pdm09 and B/Yamagata viruses as well as some A/H3N2 viruses were detected during the 2017–2018 season (Figure 2B).

### Influenza-related hospital contacts and contact rates

**Figure 3:**
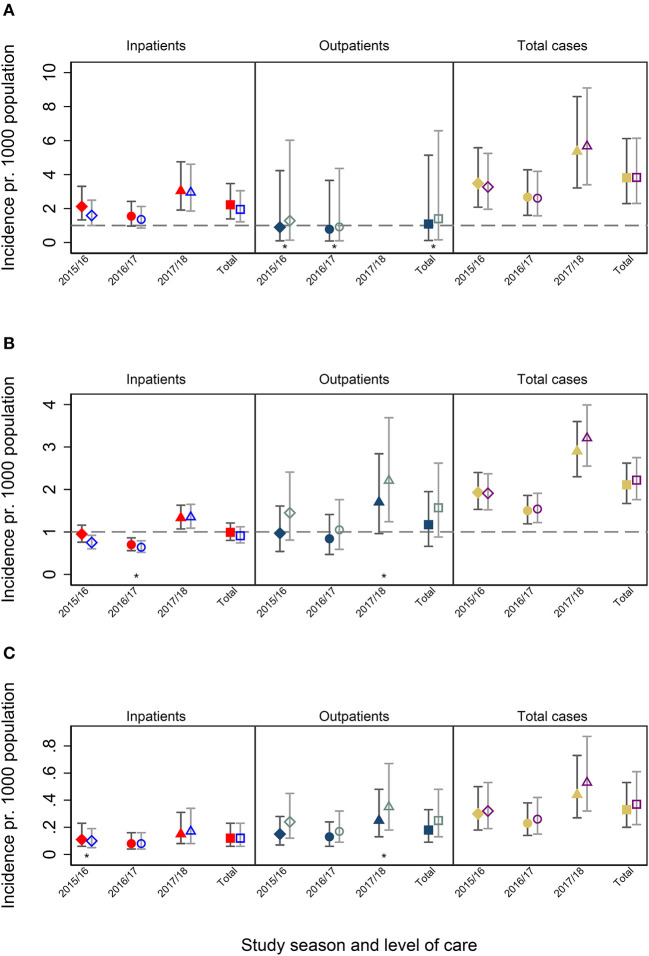
**(A)** Incidence among patients <1 years of age. **(B)** Incidence among patients 1–5 years of age. **(C)** Incidence among patients 6–18 years of age. Filled symbols represent national estimates, hollow symbols represent the population living in the catchment area of hospitals participation in NorEpis. 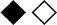: Season 2015/16 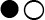: Season 2016–17 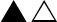: Season 2017/18 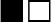: Total of seasons. Filled red and hollow blue symbols indicate inpatients. Filled dark blue and hollow gray symbols indicate outpatients. Filled yellow and hollow purple symbols indicate in- and outpatients combined. ^*^Low number of observations when calculating the probability that a patient is influenza positive given it has one of the ICD-10 codes (95% CI). A one-sided, 97.5% confidence interval was used for calculating the proportions used for the incidence calculation.

The ICD-10 codes from the final discharge diagnoses were retrieved for 307 of 309 influenza-positive cases and for 2,636 of 2,701 influenza-negative cases among the study participants (Supplementary Table 3). In the study population, no children had more than three hospital contacts with an influenza-specific diagnosis registered for 21 days following enrollment (Supplementary Figure 1). We estimated the proportion of study participants with confirmed influenza who received any of the ICD-10 codes J09–J11.1 and the proportion of study participants with these codes who tested positive for influenza (Supplementary Table 4). Using these proportions, we estimated that, on average, 1.06 (range 0.88–1.28) hospital-attended influenza cases per 1,000 children occurred in the catchment area of the study hospitals during the study period, which corresponds to 0.96 (0.80–1.16) hospital-attended influenza cases per 1,000 children <18 years of age nationally each year (Table 2). The highest incidence was found among the youngest children, with 3.8 (range 2.3–6.1) hospital-attended influenza cases per 1,000 children <1 year of age. National estimates of hospital-attended influenza and estimates for the study hospitals were similar for all age groups (Figure 3).

**Table 2:** Estimated influenza incidence per 1,000 in children 0–18 years of age.

	**Study season**	**Hospitals catchment area**	**Norway**
Outpatient cases	2015/16	0.60 (0.32–1.07)	0.39 (0.20–0.69)
	2016/17	0.52 (0.19–1.20)	0.39 (0.15–0.91)
	2017/18	1.26 (0.53–2.48)	0.93 (0.39–1.83)
	Total	0.74 (0.48–1.09)	0.52 (0.34–0.77)
Inpatient cases	2015/16	0.41 (0.31–0.53)	0.49 (0.37–0.64)
	2016/17	0.31 (0.18–0.52)	0.32 (0.19–0.53)
	2017/18	0.59 (0.42–0.82)	0.55 (0.39–0.77)
	Total	0.45 (0.37–0.54)	0.46 (0.38–0.56)
Total	2015/16	0.94 (0.71–1.21)	0.91 (0.69–1.17)
	2016/17	0.77 (0.48–1.22)	0.72 (0.44–1.13)
	2017/18	1.44 (1.02–1.99)	1.24 (0.87–1.71)
	Total	1.06 (0.88–1.28)	0.96 (0.80–1.16)

### Risk groups

Among 309 influenza cases in study participants, 21% had one or more risk conditions according to information recorded in the national registries (Table 3). Asthma and wheezing were the most common risk conditions reported, with ~16% of the confirmed influenza cases having received an ICD-10 or ICDC-2 code for asthma prior to enrollment. This was followed by premature birth (~11%) and congenital heart disease (~7%). When comparing the estimated incidence rates in risk groups to the overall study population, considerably larger incidence rates were observed in all risk groups (Table 3), ranging from 1.3 (range 1.1–1.6) per 1,000 children with epilepsy to 17.0 (14.1–20.5) per 1,000 in children with bronchopulmonary dysplasia (Table 3). However, when comparing children with the combination of any of trisomy 21, congenital heart disease, pulmonary disease, bronchopulmonary dysplasia, immunosuppression, cancer, asthma and epilepsy to children without these diagnoses, no significant associated with influenza was evident after adjusting for age [unadjusted OR 1.5 (1.1–2.0), adjusted OR 0.9 (0.6–1.3)].

**Table 3:** Number of participants in high-risk groups of influenza, and mean annual rate of hospital contact per 1,000 children by risk-groups in children under 18 years of age in Norway, 2015–2017.

Risk-group*	**NorEPIS**		**Norway, 2015–2017**		
	**Hospital**	**Influenza**	**Mean population**	**Estimated number**	**Mean annual**
	**contacts†**	**cases^‡^**	**size (0–18 years)**	**of influenza cases**	**annual**
	***N* = 3,010 (%)**	***N* = 309 (%)**	**in riskgroups^§^**	**treated in hospitals^||^**	**rate**
Prematurity, gestational age <37 weeks	326 (10.8)	35 (11.3)	63,374	123.3 (102.0–148.1)	1.9 (1.6–2.3)
Trisomy 21	32 (1.1)	1 (0.3)	1,638	3.5 (2.9–4.2)	2.1 (1.8–2.6)
Congenital heart disease	174 (5.8)	23 (7.4)	18,096	81.0 (67.0–97.2)	4.5 (3.7–5.4)
Pulmonary disease^¶^	46 (1.5)	5 (1.6)	2,363	17.6 (14.6–21.2)	7.5 (6.2–9.0)
Bronchopulmonary dysplasia	63 (2.1)	5 (1.6)	1,034	17.6 (14.6–21.2)	17.0 (14.1–20.5)
Immunosuppression/deficiency	32 (1.1)	6 (1.9)	1,888	21.1 (17.5–25.4)	11.2 (9.2–13.4)
Cancer**	21 (0.7)	3 (1.0)	21,14	10.6 (8.7–12.7)	5.0 (4.1–6.0)
Asthma	265 (8.8)	48 (15.5)	55,268	169.0 (139.8–203.0)	3.1 (2.5–3.7)
Nevromuscular disease	92 (3.1)	17 (5.5)	10,809	59.9 (49.5–71.9)	5.5 (4.6–6.7)
Epilepsy	14 (0.5)	4 (1.3)	10,690	14.0 (11.6–16.9)	1.3 (1.1–1.6)
Any underlying condition^‡^	471 (15.7)	65 (21.0)	84,776	229.0 (189.4–275.0)	2.7 (2.2–3.2)

^*^ICD-10 codes used to identify risk groups are provided in supplementary data. †Total number of hospital contacts in the NorEPIS cohort.^‡^Number of influenza cases in the NorEPIS cohort. § Mean size of background population during the 3 study years. || Estimated mean annual number of influenza cases treated at Norwegian hospitals. ¶ Pulmonary disease not including asthma or bronchopulmonary dysplasia. ^**^Cancer patients receiving active chemotherapeutic treatment. ††Diagnosed with any of Trisomy 21, congenital heart disease, pulmonary disease, bronchopulmonary dysplasia, immunosuppression, cancer, asthma, epilepsy.

Among children with confirmed influenza, those with congenital heart disease were more likely to receive inpatient treatment. Prematurity and neuromuscular disease were also positively associated with inpatient admission in the univariate analyses; however, the association was not significant in the multivariate analysis including age group, hospital, study season, and month of hospital contact as independent variables (Table 4).

**Table 4:** Logistic regression comparing influenza positive outpatient and inpatient cases.

	**Level of care**					
	**Outpatient (%)**	**Inpatient (%)**	**OR (95% CI)**	***P***	aOR (95% CI)*	***P***
**Age group**
<6 m	13 (43.3)	17 (56.7)	Ref.		Ref.	
6–11 m	13 (39.4)	20 (60.6)	1.18 (0.43–3.21)	0.75	1.44 (0.47–4.40)	0.52
1–2 y	64 (47.8)	70 (52.2)	0.84 (0.38–1.86)	0.66	0.84 (0.34–2.07)	0.71
3–5 y	31 (63.3)	18 (36.7)	0.44 (0.18–1.12)	0.09	0.33 (0.11–0.98)	0.05
6–17 y	35 (57.4)	26 (42.6)	0.57 (0.24–1.37)	0.21	0.62 (0.23–1.66)	0.35
**Sex**
Male	90 (51.7)	84 (48.3)	Ref.		Ref.	
Female	66 (49.6)	67 (50.4)	1.09 (0.69–1.71)	0.72	1.11 (0.66–1.86)	0.70
**Born prematurely**
Term	145 (53.3)	127 (46.7)	Ref.		Ref.	
Premature	11 (31.4)	24(68.6)	2.49 (1.17–5.29)	0.02	2.00 (0.82–4.91)	0.13
**Downs syndrome**
No	156 (51.0)	150 (49.0)				
Yes	0 (0.0)	1 (100.0)	–	–	–	–
**Congenital heart disease**
No	151 (53.2)	133 (46.8)	Ref.		Ref.	
Yes	5 (21.7)	18 (78.3)	4.09 (1.48–11.31)	0.01	3.80 (1.20–12.02)	0.02
**Pulmonary disease6**
No	155 (51.3)	147 (48.7)	Ref.		Ref.	
Yes	1 (20.0)	4 (80.0)	4.22 (0.47–38.17)	0.20	1.70 (0.14–20.28)	0.67
**Bronchopulmonary dysplasia**
No	156 (51.7)	146 (48.3)				
Yes	0 (14.3)	5 (100.0)	–	–	–	–
**Immunosuppression/deficiency**
No	152 (50.5)	149 (49.5)	Ref.		Ref.	
Yes	4 (66.7)	2 (33.3)	0.51 (0.09–2.83)	0.44	0.13 (0.01–2.04)	0.15
**Cancer**
No	154 (50.5)	151 (49.5)				
Yes	2 (100.0)	0 (0.0)	–	–	–	–
**Asthma**
No	133 (51.4)	126 (48.7)	Ref.		Ref.	
Yes	23 (47.9)	25 (52.1)	1.15 (0.62–2.13)	0.66	1.37 (0.65–2.89)	0.41
**Nevromuscular disease**
No	152 (52.2)	139 (47.8)	Ref.		Ref.	
Yes	4 (25.0)	12 (75.0)	3.28 (1.03–10.41)	0.04	3.80 (0.94–15.39)	0.06
**Epilepsy**
No	156 (51.5)	144 (48.5)				
Yes	0 (0.0)	4 (100.0)	–	–	–	–
**Any underlying disorder** ^†^
No	129 (53.1)	114 (46.9)	Ref.		Ref.	
Yes	27 (42.2)	37 (57.8)	1.55 (0.89–2.70)	0.12	1.66 (0.85–3.24)	0.14
**Valid influenza vaccine**
No	152 (50.3)	150 (49.7)	Ref.			
Yes	4 (80.0)	1 (20.0)	0.25 (0.03–2.29)	0.22	–	–

^*^ Results are adjusted by including age group, hospital, study season and month of hospital contact as independent variables. † Diagnosed with any of Trisomy 21, congenital heart disease, pulmonary disease, bronchopulmonary dysplasia, immunosuppression, cancer, asthma, epilepsy.

## Discussion

We estimated hospital incidence rates of influenza in children <18 years of age in a large prospective hospital cohort study in Norway. We observed that ~10% of pediatric hospital contacts among children with respiratory infections or fever could be attributed to influenza, a proportion that is similar to that seen in other studies (7, 32). The highest incidence of influenza was found in the first year of life, and the relative contribution of influenza to the number of hospital-attended children with acute respiratory disease increased with age.

The majority of children with acute respiratory infections were <5 years of age, and almost 50% were <1 year of age. Among hospital contacts caused by respiratory symptoms or fever in children, the proportion attributable to influenza increased from 3% in children <6 months of age to 25% in children 6–18 years of age. It is likely that the threshold to seek medical treatment and hospital care increases with age (5), contributing to the disproportionate age distribution. Moreover, other respiratory viruses such as respiratory syncytial virus and human metapneumovirus contribute more to the disease burden in young children and often cause more severe disease compared to influenza (33–36), further augmenting the disproportionate distribution of the age groups.

The age-specific incidence rates in our study are within a previously reported range for annual pediatric influenza hospitalizations in other industrialized countries (3, 4, 9–11). However, the average incidence rate of 0.96 influenza cases per 1,000 children <18 years of age found in this study is considerably higher than the ~0.29 influenza cases per 1,000 children previously reported in children <20 years of age in Norwegian hospitals (4). This earlier study was registry-based and derived incidence estimates from the use of influenza-specific ICD-10 codes from the NPR. The strength of our study is that incidence rates were calculated based on a linkage of data from prospective surveillance with laboratory confirmation, together with national registry-based hospital data. We found that a higher percentage of influenza-positive inpatients (63.6%) than outpatient cases (22.4%) was assigned influenza-specific ICD-10 codes in our study. Interestingly, ~35% of confirmed influenza cases among inpatient cases had not received influenza-specific ICD-10 codes, although the longer duration of stay for inpatient cases allows for laboratory analysis to be completed before the patient is diagnosed. Using influenza-specific ICD-10 codes, registry data alone would likely lead to underestimating the true hospital burden, as most outpatients are missed.

It is possible that the proportion of cases without an influenza ICD-10 code decreased in the participating hospitals during the study. This could especially be true for outpatient cases, as testing activity could increase as a result of the study. If the proportion of influenza patients without ICD-10 codes for influenza is smaller in study hospitals compared to other hospitals, this could cause the national influenza incidence to be underestimated. Outpatients rarely receive influenza-specific codes either due to a delay in test results or a lack of testing. Only 22% of influenza-positive outpatients in the study received an influenza-specific ICD-10 code compared to 64% of inpatients; therefore, we believe the increased testing activity and the tendency to use influenza-specific ICD-10 codes was a minor concern.

### Risk groups

Among children with confirmed influenza, only those with congenital heart disease was associated with an increased risk of inpatient treatment. In other studies, pediatric patients with other risk conditions also had a greater risk of hospitalization (5, 19, 20). However, the limited number of influenza cases in each risk group in our study clearly limits the power to detect a true difference. Furthermore, participants were primarily children <5 years of age. Asthma is notoriously difficult to diagnose in the youngest children, who could receive an incorrect asthma diagnosis after other obstructive episodes (37, 38). This could erroneously increase the numbers of participants with asthma, inflate its incidence, and obscure real associations between asthma and inpatient treatment. The ~1.3–17-fold higher incidence of hospital-attended influenza among risk groups included in the study compared with the general pediatric population is in line with previous studies that demonstrated a strong association between such risk groups and influenza (5, 18). The incidence rates among children with pre-existing medical conditions were high in our study; however, the majority of the influenza burden occurred in children with no previously recognized pre-existing medical conditions.

Sex influenced neither the probability of testing positive for influenza nor the probability of inpatient admission. These results are supported by a previous study in Norway, which reported that 52% of patients with influenza-specific ICD-10 codes were female (4). We found a considerable variation in the proportion of influenza-positive cases and circulating influenza strains between the study seasons. However, the seasonal profile of viral strains matched those found during routine surveillance in Norway (21–23), indicating that the viruses encountered in the study population were representative for circulating influenza viruses in Norway during the study period.

The number of influenza-vaccinated children in this study was too small to conduct a meaningful analysis of vaccine effectiveness. Throughout the study, only 1% of the children were vaccinated against influenza, reflecting the low influenza vaccination coverage among children in Norway, with only 5,093 influenza vaccine doses distributed for the Norwegian population of ~1,100,000 children aged 0–17 during the 2017–2018 season (23).

This study, conducted in an interpandemic setting, found that most children receiving hospital treatment for seasonal influenza in Norway, were healthy without any identified underlying disorders. Older, hospital-attended, children were more likely to be diagnosed with influenza compared to younger children, who were more likely to have other respiratory viruses. Simultaneously, Norwegian children are infrequently vaccinated against influenza, and the treatment of influenza uses valuable hospital resources. Interventions against seasonal influenza could be beneficial not only for children with underlying medical conditions but also for otherwise healthy young children. This knowledge calls for an increased awareness about influenza in children, and implementation of practical public health activities such as vaccination and information campaigns on how to limit disease transmission could be considered.

## Data availability statement

The raw data supporting the conclusions of this article will be made available by the authors, without undue reservation.

## Ethics statement

The studies involving human participants were reviewed and approved by Regional Committees for Medical and Health Research Ethics, South-East A (2015/956). Written informed consent to participate in this study was provided by the participants' legal guardian/next of kin.

## The Norwegian Enhanced Pediatric Immunization Surveillance (NorEPIS) Network

Håkon Bøås (Norwegian Institute of Public Health), Terese Bekkevold (Norwegian Institute of Public Health), Liliana Vázquez Fernández (Norwegian Institute of Public Health), Olav Hungnes (Norwegian Institute of Public Health), Anne-Marte Bakken Kran (Norwegian Institute of Public Health/Oslo University Hospital Ullevål), Elmira Flem (Formerly Norwegian Institute of Public Health), Lise Beier Havdal (Akershus University Hospital), Christopher Inchley (Akershus University Hospital), Truls Michael Leegaard (Akershus University Hospital), Britt Nakstad (University of Oslo/Formerly Akershus University Hospital), Astrid Elisabeth Rojahn (Oslo University Hospital Ullevål), Ketil Størdal (Østfold Hospital), Sara Debes (Østfold Hospital), Henrik Døllner (St. Olavs University Hospital/Norwegian University of Science and Technology), Svein Arne Nordbø (St. Olavs University Hospital/Norwegian University of Science and Technology), Bjørn Barstad (Stavanger University Hospital), Elisebet Haarr (Stavanger University Hospital).

## Author contributions

EF drafted the study protocol. EF, TB, and HB coordinated the study. HB, TB, LH, A-MK, AR, KS, SD, HD, SN, BB, EH, BN, TL, OH, and EF contributed directly to the acquisition of data. HB, TB, LF, and LH contributed to data cleaning, verification and preparation. HB, TB, LF, and A-MK had access to the final linked dataset. HB conducted the statistical analysis with support from LH. HB drafted the manuscript with support from LH. All authors were involved in the conceptualization of the study, contributed to the interpretation of the results, contributed to the revision of the manuscript, and approved the final version for submission.

## Funding

This work was supported by the Research Council of Norway (240207/F20). The study sponsor had no saying in study design, collection, analysis, or interpretation of data, the writing of the paper or the decision to submit the paper for publication.

## Conflict of interest

Author EF is currently employed by Merck Sharp & Dohme Corp., Drammen, Norway, a subsidiary of Merck & Co., Inc., Kenilworth, NJ. The work for the current study was conducted by EF under the previous affiliation. The remaining authors declare that the research was conducted in the absence of any commercial or financial relationships that could be construed as a potential conflict of interest.

## Publisher's note

All claims expressed in this article are solely those of the authors and do not necessarily represent those of their affiliated organizations, or those of the publisher, the editors and the reviewers. Any product that may be evaluated in this article, or claim that may be made by its manufacturer, is not guaranteed or endorsed by the publisher.

## Author disclaimer

Data from the Norwegian Patient Registry have been used in this publication. The interpretation and reporting of these data are the sole responsibility of the authors, and no endorsement by the Norwegian Patient Registry is intended nor should be inferred.
