# Supplementary material for: The burden of hospital-attended influenza in Norwegian children
Source: Front Pediatr. 2022 Sep 7;10:963274. doi: 10.3389/fped.2022.963274 (PMC9491848; doi:10.3389/fped.2022.963274)
Supplement: Supplementary file 2 [file Table_2.DOCX]

Supplemental table 2: Variables retrieved from national health registries

| **NPR** | **KUHR** | **The Norwegian Prescription Database (NorPD)** | **Norwegian Immunisation Registry (SYSVAK)** | **Medical Birth Registry of Norway** |
| --- | --- | --- | --- | --- |
| **Variables** | | | | |
| Date of hospital contact | Age | Year of death | Vaccine code | The child's weight |
| Discharge date | ICPC-2 Codes | Month of death | Vaccination Date | Gestational age |
| Duration of hospital stay | Type of consultation (General practition or “legevakt”) | Project-specific serial number for the individual prescription (delivery)) | Product-specific name | Asthma |
| Level of care (inn-patient, day-treatment or polyclinical treatment) | Consultation week/month/year | Week of delivery | Batch number | Does not consent to register information about smoking habits |
| Level of emergency at arrival | Specifies the cost for the patient. | Month of delivery |  | Smoking habits before the pregnancy |
| The patients condition at discharge | Tariff codes (cost) | Year of delivery |  | Cigarettes pr. Day before the pregnancy |
| Number of days |  | The number of packs of the prescription |  | Smoking habits at the end of the pregnancy |
| ICD-10 diagnostic codes |  | The prescription's total number of defined daily doses (DDD) |  | Cigarettes pr. day at the end of the pregnancy |
| Diagnosis related groups (DRG) |  | Prescription category |  | Length of the child |
| Weighted DRGs (Estimated resource use throughout the stay based on average costs.) |  | ATC codes for Antibacterial agents for systemic use, Antiviral agents for systemic use and Palivizumab |  | Z-score |
| Corrected DRG-point |  | Defined daily doses (DDD) for ATC code |  | Vaginal birth |
| Surgical or medical DGR |  | Unit of measure for defined daily dose (DDD) |  | Cesarean section performed |
| Complicating DRG |  | Drug class (Prescription free/prescription drugs/narcotic drugs/Other addictive drugs) |  | Planned cesarean section |
| Specific DRG |  |  |  | Placenta weight |
| Main diagnosis in DRG |  |  |  | Child’s health (ICD-10 diagnosis) at birth |
| ATC codes (during hospital stay) |  |  |  | Respiratory distress syndrome |
| County of residence |  |  |  | Treated by respirator |
| Region of residence |  |  |  | CPAP |
| Organization number and name of hospital |  |  |  | Congenital malformation |
|  |  |  |  | Congenital heart disease |
|  |  |  |  | chromosomal aberrations |
|  |  |  |  | Downs syndrome |
|  |  |  |  | Parity |
|  |  |  |  | Plurality |
|  |  |  |  | Mothers age |
|  |  |  |  | Year of death (Child) |
|  |  |  |  | Month of death (Child) |
