# Supplementary material for: The burden of hospital-attended influenza in Norwegian children
Source: Front Pediatr. 2022 Sep 7;10:963274. doi: 10.3389/fped.2022.963274 (PMC9491848; doi:10.3389/fped.2022.963274)
Supplement: Supplementary file 3 [file Table_3.DOCX]

Supplemental table 3: The number of acute hospital contacts registered among the NorEPIS study participants, and the number of acute disease episodes in children < 18 years registered in the Norwegian patient registry with an ICD-10 diagnosis of J09.x, J10.0, J10.1, J10.8, J11.0 or J11.1

|  | **Study year** | **Outpatient contacts** | **Inpatient contacts** | **Total** |
| --- | --- | --- | --- | --- |
| **Number of hospital contacts at recruitment** |  | **Positive (n=156) / Negative (n=1030)** | **Positive (n=151) / Negative (n=1606)** | **Positive (n=307) / Negative (n=2636)** |
|  | 2015/16 (n=66) | 18/3 | 43/2 | 61/5 |
|  | 2016/17 (n=33) | 8/1 | 20/4 | 28/5 |
|  | 2017/18 (n=45) | 9/0 | 33/3 | 42/3 |
|  | Total (n=144) | 35/4 | 96/9 | 131/13 |
| **Number of registered disease episodes in NPR in study participants*.** |  | **Positive (n=154) / Negative (n=1029)** | **Positive (n=153) / Negative (n=1607)** | **Positive (n=307) / Negative (n=2636)** |
|  | 2015/16 (n=74) | 20/4 | 47/3 | 67/7 |
|  | 2016/17 (n=37) | 10/2 | 20/5 | 30/7 |
|  | 2017/18 (n=48) | 10/0 | 33/5 | 43/5 |
|  | Total (n=159) | 40/6 | 100/13 | 140/19 |
|  | **Study year** | **Outpatient contacts** | **Inpatient contacts** | **Total** |
| **All disease episodes registered in NPR in study hospitals**^†^**.** | 2015/16 | 90 | 121 | 211 |
|  | 2016/17 | 65 | 103 | 168 |
|  | 2017/18 | 136 | 215 | 351 |
|  | Total | 291 | 439 | 730 |
| **All disease episodes registered in NPR from all Norwegian hospitals**^†^**.** | 2015/16 | 147 | 374 | 521 |
|  | 2016/17 | 127 | 273 | 400 |
|  | 2017/18 | 255 | 515 | 770 |
|  | Total | 529 | 1162 | 1691 |
| * Any contact registered in the 21 days around recruitment is considered as one episode  † All contacts registered in the 21 days from first contact is considered as one episode | | | | |
