# Supplementary material for: The burden of hospital-attended influenza in Norwegian children
Source: Front Pediatr. 2022 Sep 7;10:963274. doi: 10.3389/fped.2022.963274 (PMC9491848; doi:10.3389/fped.2022.963274)
Supplement: Supplementary file 4 [file Table_4.DOCX]

Supplemental table 4: Total probability over three seasons that an influenza patient will be diagnosed with any of the ICD-10 codes J09.x, J10.0, J10.1, J10.8, J11.0 or J11.1, and the probability that a patient diagnosed with any of these codes are influenza positive. Any contact registered in the Norwegian Patient Registry during the 21 days around recruitment is considered as one episode

| **Age** | **Level of treatment** | **Probability (%) that an influenza patient will be given the selected ICD-10 codes (95% CI)** | **Probability (%) that patient is influenza positive given it has one of the ICD-10 codes (95% CI)** |  |  |
| --- | --- | --- | --- | --- | --- |
| **0-1** | **Outpatient** | 12 (2.5 - 31.2) | 100 (29.2 - 100)* |  |  |
|  | **Inpatient** | 63.2 (46.0 - 78.2) | 82.8 (64.2 - 94.2) |  |  |
|  | **Total** | 41.3 (29.0 - 54.4) | 83.9 (66.3 - 94.5) |  |  |
| **1-5** | **Outpatient** | 24.5 (16.2 - 34.4) | 88.5 (69.8 - 97.6) |  |  |
|  | **Inpatient** | 69.7 (59.0 - 79.0) | 95.4 (87.1 - 99.0) |  |  |
|  | **Total** | 46.4 (39.1 - 54.0) | 93.4 (86.2 - 97.5) |  |  |
| **6-18** | **Outpatient** | 42.9 (26.3 -60.6) | 83.3 (58.6 - 96.4) |  |  |
|  | **Inpatient** | 53.8 (33.4 - 73.4) | 73.7 (48.8 - 90.9) |  |  |
|  | **Total** | 47.5 (34.6 - 60.7) | 78.4 (61.8 - 90.2) |  |  |
| **0-18** | **Outpatient** | 26.0 (19.2 - 33.6) | 87.0 (73.7 - 95.1) |  |  |
|  | **Inpatient** | 65.4 (57.3 - 72.9) | 88.5 (81.1 - 93.7) |  |  |
|  | **Total** | 45.6 (39.9 - 51.4) | 88.1 (82.0 - 92.6) |  |  |
| *Low number of participants, a one-sided 97,5% CI was used | | | | | |
