# Supplementary material for: The burden of hospital-attended influenza in Norwegian children
Source: Front Pediatr. 2022 Sep 7;10:963274. doi: 10.3389/fped.2022.963274 (PMC9491848; doi:10.3389/fped.2022.963274)
Supplement: Supplementary file 5 [file Table_5.DOCX]

### Supplementary information

### Inclusion criteria:

Participants in the NorEPIS respiratory study meet the following inclusion criteria:

1. Children < 18 years of age

2. Residents of surveillance areas admitted to participating hospital within 48 hours prior to enrolment with:

1. Children 1-18 years of age with measured fever of ≥ 38⁰C or a history of fever of ≥ 38⁰C with onset within the past 10 days.
2. Children <1 year of age without fever, or with onset of fever within the past 10 days, and at least one acute respiratory sign or symptom including but not limited to cough, sore throat, nasal discharge, apnea, tachypnea, respiratory distress, wheezing, stridor, otitis media or earache, sinusitis, sepsis-like illness, dehydration, feeding difficulties, impaired general condition, vomiting, febrile seizures, meningitis, encephalitis.

### Exclusion criteria:

Exclusion criteria for the NorEPIS respiratory study is:

- Persons above 18 years of age
- Children residing outside the surveillance areas
- Children not enrolled within 48 hours of admission
- Newborns who never left the hospital
- Children transferred from another hospital
- Children admitted for elective hospitalization, injury or social indication
