# Supplementary figures and images for: The burden of hospital-attended influenza in Norwegian children
Source: Front Pediatr. 2022 Sep 7;10:963274. doi: 10.3389/fped.2022.963274 (PMC9491848; doi:10.3389/fped.2022.963274)

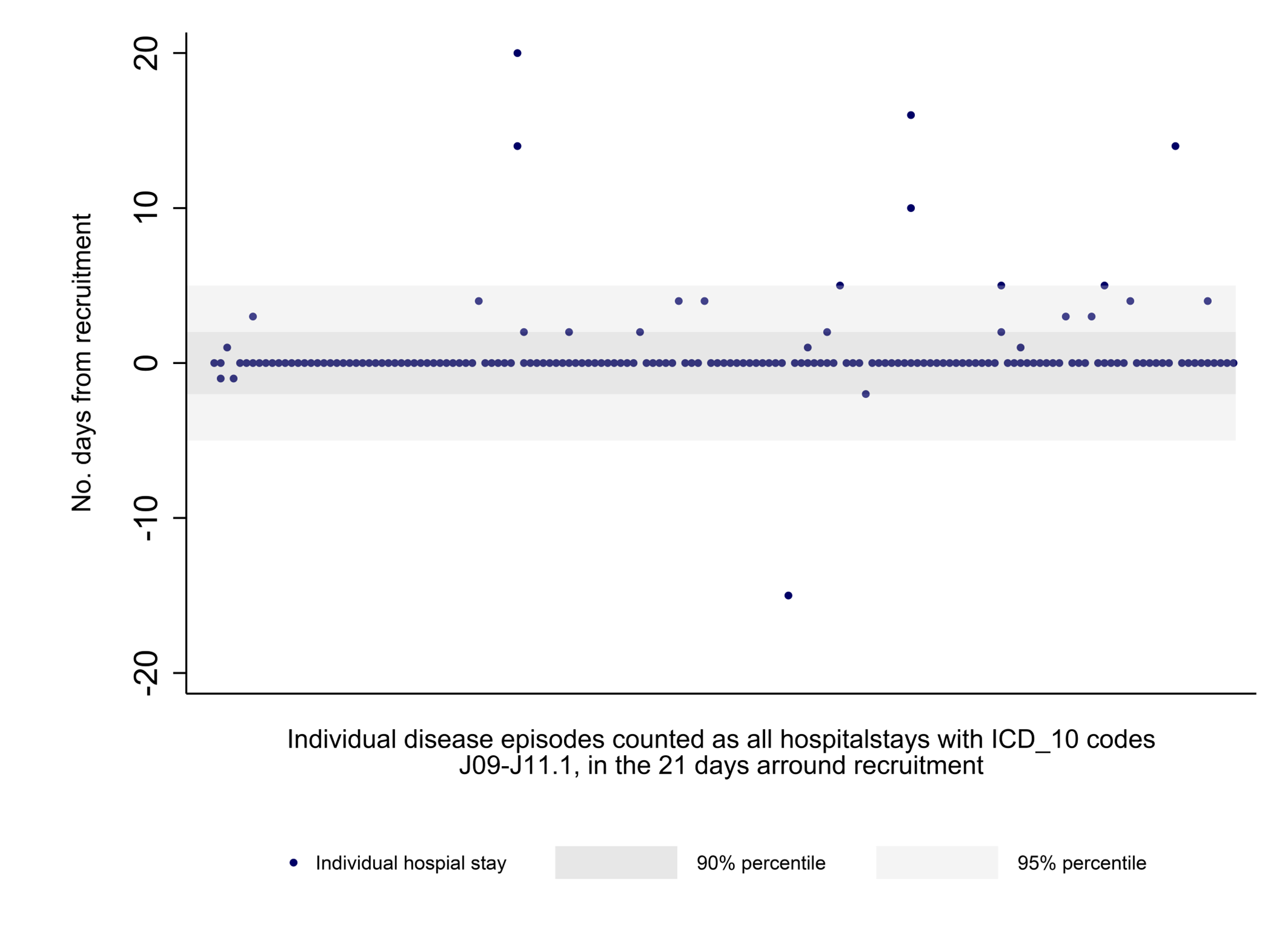

Supplement: Supplementary file 6 [file Image_1.TIFF]
